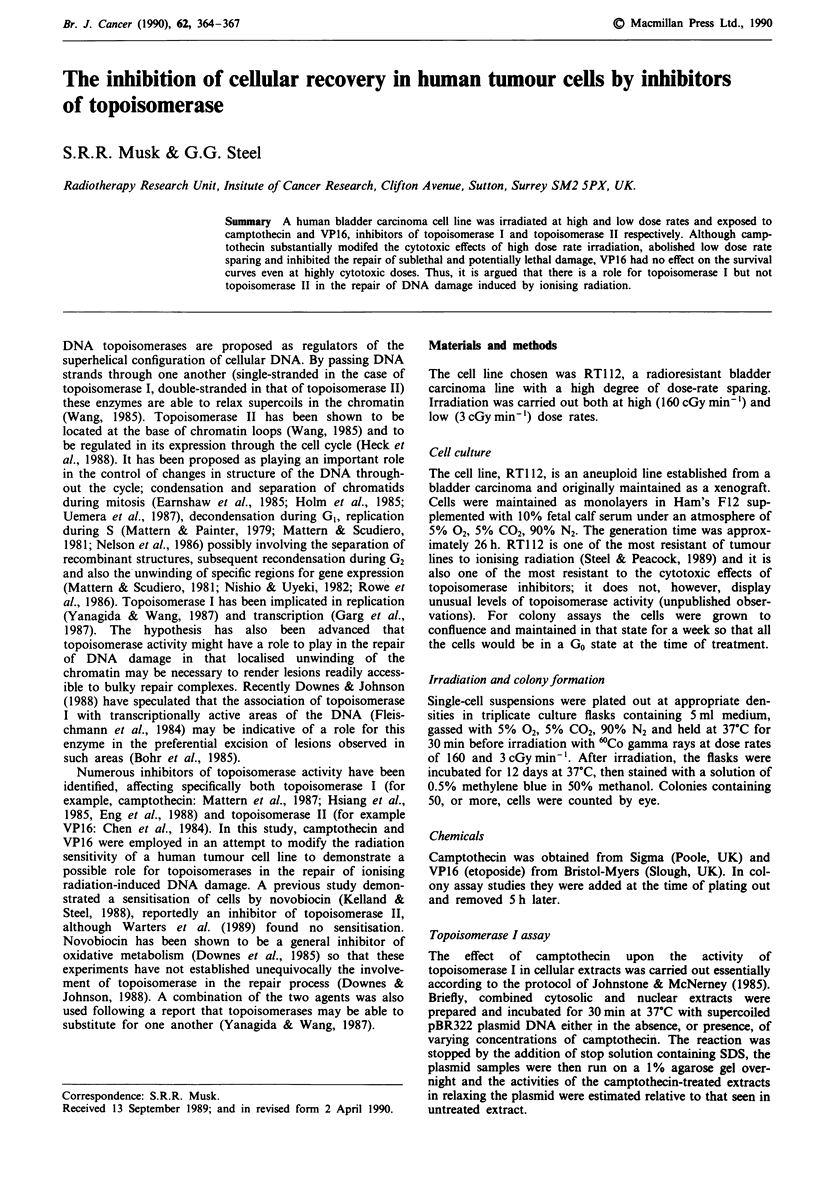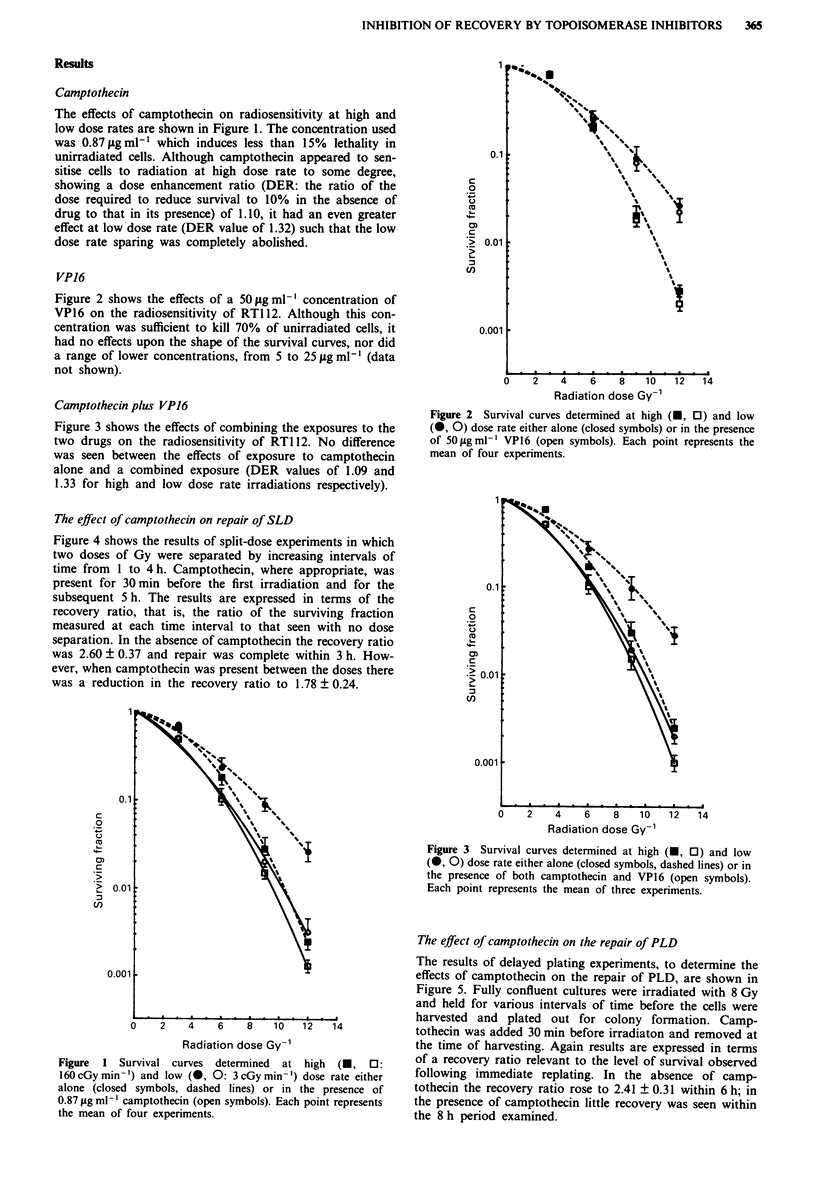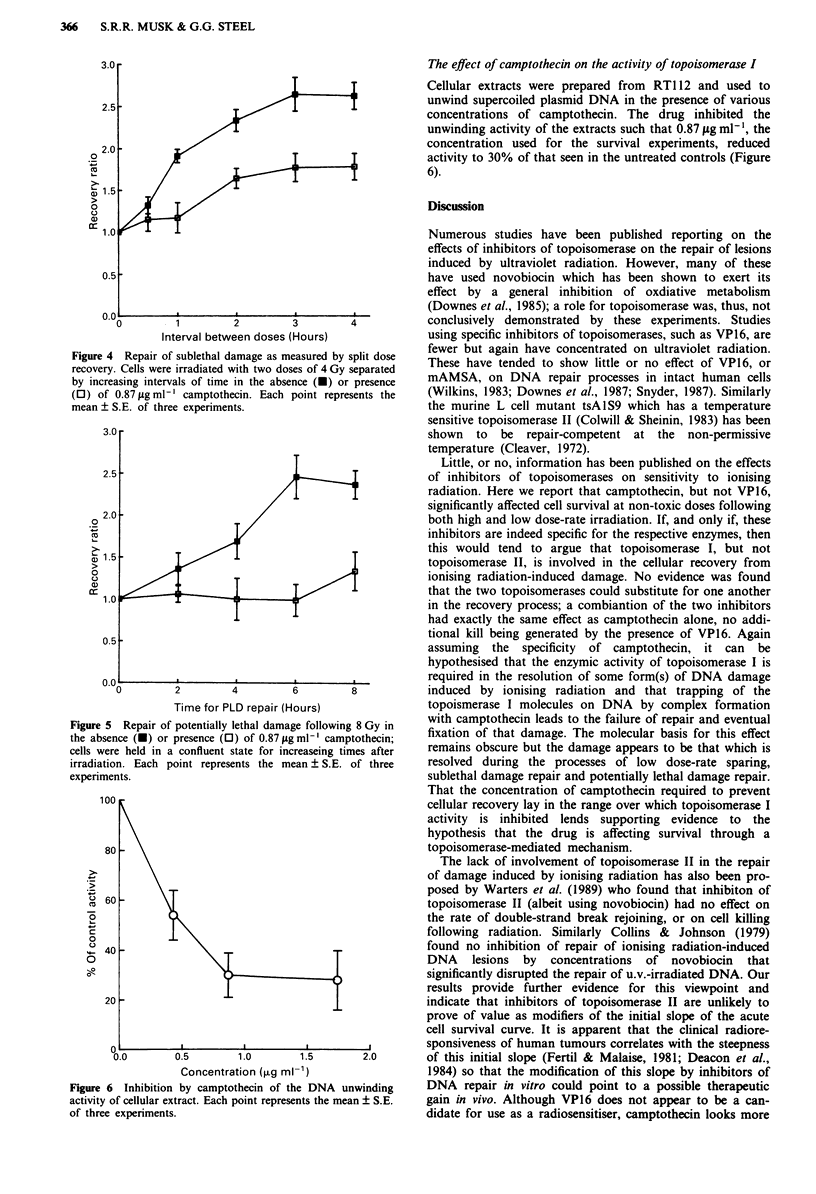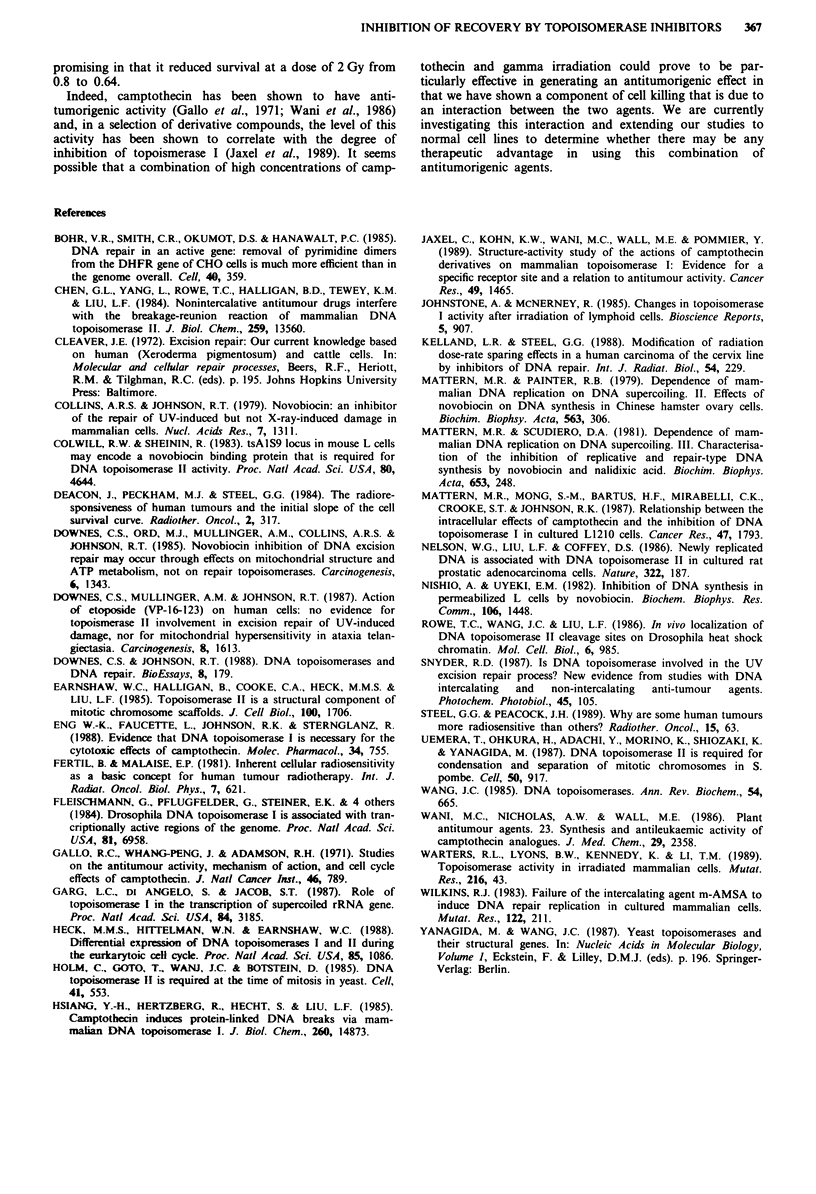# The inhibition of cellular recovery in human tumour cells by inhibitors of topoisomerase.

**DOI:** 10.1038/bjc.1990.298

**Published:** 1990-09

**Authors:** S. R. Musk, G. G. Steel

**Affiliations:** Radiotherapy Research Unit, Institute of Cancer Research, Sutton, Surrey, UK.

## Abstract

A human bladder carcinoma cell line was irradiated at high and low dose rates and exposed to camptothecin and VP16, inhibitors of topoisomerase I and topoisomerase II respectively. Although camptothecin substantially modified the cytotoxic effects of high dose rate irradiation, abolished low dose rate sparing and inhibited the repair of sublethal and potentially lethal damage, VP16 had no effect on the survival curves even at highly cytotoxic doses. Thus, it is argued that there is a role for topoisomerase I but not topoisomerase II in the repair of DNA damage induced by ionising radiation.


Br. J. Cancer (1990), 62, 364-367 © Macmillan Press Ltd., 1990

### The inhibition of cellular recovery in human tumour cells by inhibitors of topoisomerase

S.R.R. Musk & G.G. Steel

*Radiotherapy Research Unit, Insitute of Cancer Research, Clifton Avenue, Sutton, Surrey SM2 5PX, UK.*

**Summary** A human bladder carcinoma cell line was irradiated at high and low dose rates and exposed to camptothecin and VP16, inhibitors of topoisomerase I and topoisomerase II respectively. Although camptothecin substantially modifed the cytotoxic effects of high dose rate irradiation, abolished low dose rate sparing and inhibited the repair of sublethal and potentially lethal damage, VP16 had no effect on the survival curves even at highly cytotoxic doses. Thus, it is argued that there is a role for topoisomerase I but not topoisomerase II in the repair of DNA damage induced by ionising radiation.

DNA topoisomerases are proposed as regulators of the superhelical configuration of cellular DNA. By passing DNA strands through one another (single-stranded in the case of topoisomerase I, double-stranded in that of topoisomerase II) these enzymes are able to relax supercoils in the chromatin (Wang, 1985). Topoisomerase II has been shown to be located at the base of chromatin loops (Wang, 1985) and to be regulated in its expression through the cell cycle (Heck *et al*., 1988). It has been proposed as playing an important role in the control of changes in structure of the DNA throughout the cycle; condensation and separation of chromatids during mitosis (Earnshaw *et al*., 1985; Holm *et al*., 1985; Uemera *et al*., 1987), decondensation during $G_1$, replication during S (Mattern & Painter, 1979; Mattern & Scudiero, 1981; Nelson *et al*., 1986) possibly involving the separation of recombinant structures, subsequent recondensation during $G_2$ and also the unwinding of specific regions for gene expression (Mattern & Scudiero, 1981; Nishio & Uyeki, 1982; Rowe *et al*., 1986). Topoisomerase I has been implicated in replication (Yanagida & Wang, 1987) and transcription (Garg *et al*., 1987). The hypothesis has also been advanced that topoisomerase activity might have a role to play in the repair of DNA damage in that localised unwinding of the chromatin may be necessary to render lesions readily accessible to bulky repair complexes. Recently Downes & Johnson (1988) have speculated that the association of topoisomerase I with transcriptionally active areas of the DNA (Fleischmann *et al*., 1984) may be indicative of a role for this enzyme in the preferential excision of lesions observed in such areas (Bohr *et al*., 1985).

Numerous inhibitors of topoisomerase activity have been identified, affecting specifically both topoisomerase I (for example, camptothecin: Mattern *et al*., 1987; Hsiang *et al*., 1985, Eng *et al*., 1988) and topoisomerase II (for example VP16: Chen *et al*., 1984). In this study, camptothecin and VP16 were employed in an attempt to modify the radiation sensitivity of a human tumour cell line to demonstrate a possible role for topoisomerases in the repair of ionising radiation-induced DNA damage. A previous study demonstrated a sensitisation of cells by novobiocin (Kelland & Steel, 1988), reportedly an inhibitor of topoisomerase II, although Warters *et al*. (1989) found no sensitisation. Novobiocin has been shown to be a general inhibitor of oxidative metabolism (Downes *et al*., 1985) so that these experiments have not established unequivocally the involvement of topoisomerase in the repair process (Downes & Johnson, 1988). A combination of the two agents was also used following a report that topoisomerases may be able to substitute for one another (Yanagida & Wang, 1987).

Correspondence: S.R.R. Musk.
Received 13 September 1989; and in revised form 2 April 1990.

### Materials and methods

The cell line chosen was RT112, a radioresistant bladder carcinoma line with a high degree of dose-rate sparing. Irradiation was carried out both at high (160 cGy $min^{-1}$) and low (3 cGy $min^{-1}$) dose rates.

### *Cell culture*

The cell line, RT112, is an aneuploid line established from a bladder carcinoma and originally maintained as a xenograft. Cells were maintained as monolayers in Ham's F12 supplemented with 10% fetal calf serum under an atmosphere of 5% $O_2$, 5% $CO_2$, 90% $N_2$. The generation time was approximately 26 h. RT112 is one of the most resistant of tumour lines to ionising radiation (Steel & Peacock, 1989) and it is also one of the most resistant to the cytotoxic effects of topoisomerase inhibitors; it does not, however, display unusual levels of topoisomerase activity (unpublished observations). For colony assays the cells were grown to confluence and maintained in that state for a week so that all the cells would be in a $G_0$ state at the time of treatment.

### *Irradiation and colony formation*

Single-cell suspensions were plated out at appropriate densities in triplicate culture flasks containing 5 ml medium, gassed with 5% $O_2$, 5% $CO_2$, 90% $N_2$ and held at 37°C for 30 min before irradiation with $^{60}Co$ gamma rays at dose rates of 160 and 3 cGy $min^{-1}$. After irradiation, the flasks were incubated for 12 days at 37°C, then stained with a solution of 0.5% methylene blue in 50% methanol. Colonies containing 50, or more, cells were counted by eye.

### *Chemicals*

Camptothecin was obtained from Sigma (Poole, UK) and VP16 (etoposide) from Bristol-Myers (Slough, UK). In colony assay studies they were added at the time of plating out and removed 5 h later.

### *Topoisomerase I assay*

The effect of camptothecin upon the activity of topoisomerase I in cellular extracts was carried out essentially according to the protocol of Johnstone & McNerney (1985). Briefly, combined cytosolic and nuclear extracts were prepared and incubated for 30 min at 37°C with supercoiled pBR322 plasmid DNA either in the absence, or presence, of varying concentrations of camptothecin. The reaction was stopped by the addition of stop solution containing SDS, the plasmid samples were then run on a 1% agarose gel overnight and the activities of the camptothecin-treated extracts in relaxing the plasmid were estimated relative to that seen in untreated extract.

### Results

### *Camptothecin*

The effects of camptothecin on radiosensitivity at high and low dose rates are shown in Figure 1. The concentration used was 0.87 $\mu g\ ml^{-1}$ which induces less than 15% lethality in unirradiated cells. Although camptothecin appeared to sensitise cells to radiation at high dose rate to some degree, showing a dose enhancement ratio (DER: the ratio of the dose required to reduce survival to 10% in the absence of drug to that in its presence) of 1.10, it had an even greater effect at low dose rate (DER value of 1.32) such that the low dose rate sparing was completely abolished.

### *VP16*

Figure 2 shows the effects of a 50 $\mu g\ ml^{-1}$ concentration of VP16 on the radiosensitivity of RT112. Although this concentration was sufficient to kill 70% of unirradiated cells, it had no effects upon the shape of the survival curves, nor did a range of lower concentrations, from 5 to 25 $\mu g\ ml^{-1}$ (data not shown).

### *Camptothecin plus VP16*

Figure 3 shows the effects of combining the exposures to the two drugs on the radiosensitivity of RT112. No difference was seen between the effects of exposure to camptothecin alone and a combined exposure (DER values of 1.09 and 1.33 for high and low dose rate irradiations respectively).

### *The effect of camptothecin on repair of SLD*

Figure 4 shows the results of split-dose experiments in which two doses of Gy were separated by increasing intervals of time from 1 to 4 h. Camptothecin, where appropriate, was present for 30 min before the first irradiation and for the subsequent 5 h. The results are expressed in terms of the recovery ratio, that is, the ratio of the surviving fraction measured at each time interval to that seen with no dose separation. In the absence of camptothecin the recovery ratio was 2.60 ± 0.37 and repair was complete within 3 h. However, when camptothecin was present between the doses there was a reduction in the recovery ratio to 1.78 ± 0.24.

**Figure 1** Survival curves determined at high (■, □: 160 cGy min$^{-1}$) and low (●, ○: 3 cGy min$^{-1}$) dose rate either alone (closed symbols, dashed lines) or in the presence of 0.87 $\mu g\ ml^{-1}$ camptothecin (open symbols). Each point represents the mean of four experiments.

**Figure 2** Survival curves determined at high (■, □) and low (●, ○) dose rate either alone (closed symbols) or in the presence of 50 $\mu g\ ml^{-1}$ VP16 (open symbols). Each point represents the mean of four experiments.

**Figure 3** Survival curves determined at high (■, □) and low (●, ○) dose rate either alone (closed symbols, dashed lines) or in the presence of both camptothecin and VP16 (open symbols). Each point represents the mean of three experiments.

### *The effect of camptothecin on the repair of PLD*

The results of delayed plating experiments, to determine the effects of camptothecin on the repair of PLD, are shown in Figure 5. Fully confluent cultures were irradiated with 8 Gy and held for various intervals of time before the cells were harvested and plated out for colony formation. Camptothecin was added 30 min before irradiaton and removed at the time of harvesting. Again results are expressed in terms of a recovery ratio relevant to the level of survival observed following immediate replating. In the absence of camptothecin the recovery ratio rose to 2.41 ± 0.31 within 6 h; in the presence of camptothecin little recovery was seen within the 8 h period examined.

**Figure 4** Repair of sublethal damage as measured by split dose recovery. Cells were irradiated with two doses of 4 Gy separated by increasing intervals of time in the absence (■) or presence (□) of 0.87 μg ml$^{-1}$ camptothecin. Each point represents the mean ± S.E. of three experiments.

**Figure 5** Repair of potentially lethal damage following 8 Gy in the absence (■) or presence (□) of 0.87 μg ml$^{-1}$ camptothecin; cells were held in a confluent state for increaseing times after irradiation. Each point represents the mean ± S.E. of three experiments.

**Figure 6** Inhibition by camptothecin of the DNA unwinding activity of cellular extract. Each point represents the mean ± S.E. of three experiments.

*The effect of camptothecin on the activity of topoisomerase I*

Cellular extracts were prepared from RT112 and used to unwind supercoiled plasmid DNA in the presence of various concentrations of camptothecin. The drug inhibited the unwinding activity of the extracts such that 0.87 μg ml$^{-1}$, the concentration used for the survival experiments, reduced activity to 30% of that seen in the untreated controls (Figure 6).

### Discussion

Numerous studies have been published reporting on the effects of inhibitors of topoisomerase on the repair of lesions induced by ultraviolet radiation. However, many of these have used novobiocin which has been shown to exert its effect by a general inhibition of oxdiative metabolism (Downes *et al.*, 1985); a role for topoisomerase was, thus, not conclusively demonstrated by these experiments. Studies using specific inhibitors of topoisomerases, such as VP16, are fewer but again have concentrated on ultraviolet radiation. These have tended to show little or no effect of VP16, or mAMSA, on DNA repair processes in intact human cells (Wilkins, 1983; Downes *et al.*, 1987; Snyder, 1987). Similarly the murine L cell mutant tsA1S9 which has a temperature sensitive topoisomerase II (Colwill & Sheinin, 1983) has been shown to be repair-competent at the non-permissive temperature (Cleaver, 1972).

Little, or no, information has been published on the effects of inhibitors of topoisomerases on sensitivity to ionising radiation. Here we report that camptothecin, but not VP16, significantly affected cell survival at non-toxic doses following both high and low dose-rate irradiation. If, and only if, these inhibitors are indeed specific for the respective enzymes, then this would tend to argue that topoisomerase I, but not topoisomerase II, is involved in the cellular recovery from ionising radiation-induced damage. No evidence was found that the two topoisomerases could substitute for one another in the recovery process; a combiantion of the two inhibitors had exactly the same effect as camptothecin alone, no additional kill being generated by the presence of VP16. Again assuming the specificity of camptothecin, it can be hypothesised that the enzymic activity of topoisomerase I is required in the resolution of some form(s) of DNA damage induced by ionising radiation and that trapping of the topoismerase I molecules on DNA by complex formation with camptothecin leads to the failure of repair and eventual fixation of that damage. The molecular basis for this effect remains obscure but the damage appears to be that which is resolved during the processes of low dose-rate sparing, sublethal damage repair and potentially lethal damage repair. That the concentration of camptothecin required to prevent cellular recovery lay in the range over which topoisomerase I activity is inhibited lends supporting evidence to the hypothesis that the drug is affecting survival through a topoisomerase-mediated mechanism.

The lack of involvement of topoisomerase II in the repair of damage induced by ionising radiation has also been proposed by Warters *et al.* (1989) who found that inhibiton of topoisomerase II (albeit using novobiocin) had no effect on the rate of double-strand break rejoining, or on cell killing following radiation. Similarly Collins & Johnson (1979) found no inhibition of repair of ionising radiation-induced DNA lesions by concentrations of novobiocin that significantly disrupted the repair of u.v.-irradiated DNA. Our results provide further evidence for this viewpoint and indicate that inhibitors of topoisomerase II are unlikely to prove of value as modifiers of the initial slope of the acute cell survival curve. It is apparent that the clinical radioresponsiveness of human tumours correlates with the steepness of this initial slope (Fertil & Malaise, 1981; Deacon *et al.*, 1984) so that the modification of this slope by inhibitors of DNA repair *in vitro* could point to a possible therapeutic gain *in vivo*. Although VP16 does not appear to be a candidate for use as a radiosensitiser, camptothecin looks more promising in that it reduced survival at a dose of 2 Gy from 0.8 to 0.64.

Indeed, camptothecin has been shown to have anti-tumorigenic activity (Gallo *et al.*, 1971; Wani *et al.*, 1986) and, in a selection of derivative compounds, the level of this activity has been shown to correlate with the degree of inhibition of topoismerase I (Jaxel *et al.*, 1989). It seems possible that a combination of high concentrations of camptothecin and gamma irradiation could prove to be particularly effective in generating an antitumorigenic effect in that we have shown a component of cell killing that is due to an interaction between the two agents. We are currently investigating this interaction and extending our studies to normal cell lines to determine whether there may be any therapeutic advantage in using this combination of antitumorigenic agents.

### References

BOHR, V.R., SMITH, C.R., OKUMOT, D.S. & HANAWALT, P.C. (1985). DNA repair in an active gene: removal of pyrimidine dimers from the DHFR gene of CHO cells is much more efficient than in the genome overall. *Cell*, **40**, 359.

CHEN, G.L., YANG, L., ROWE, T.C., HALLIGAN, B.D., TEWEY, K.M. & LIU, L.F. (1984). Nonintercalative antitumour drugs interfere with the breakage-reunion reaction of mammalian DNA topoisomerase II. *J. Biol. Chem.*, **259**, 13560.

CLEAVER, J.E. (1972). Excision repair: Our current knowledge based on human (Xeroderma pigmentosum) and cattle cells. In: *Molecular and cellular repair processes*, Beers, R.F., Heriott, R.M. & Tilghman, R.C. (eds). p. 195. Johns Hopkins University Press: Baltimore.

COLLINS, A.R.S. & JOHNSON, R.T. (1979). Novobiocin: an inhibitor of the repair of UV-induced but not X-ray-induced damage in mammalian cells. *Nucl. Acids Res.*, **7**, 1311.

COLWILL, R.W. & SHEININ, R. (1983). tsA1S9 locus in mouse L cells may encode a novobiocin binding protein that is required for DNA topoisomerase II activity. *Proc. Natl Acad. Sci. USA*, **80**, 4644.

DEACON, J., PECKHAM, M.J. & STEEL, G.G. (1984). The radioresponsiveness of human tumours and the initial slope of the cell survival curve. *Radiother. Oncol.*, **2**, 317.

DOWNES, C.S., ORD, M.J., MULLINGER, A.M., COLLINS, A.R.S. & JOHNSON, R.T. (1985). Novobiocin inhibition of DNA excision repair may occur through effects on mitochondrial structure and ATP metabolism, not on repair topoisomerases. *Carcinogenesis*, **6**, 1343.

DOWNES, C.S., MULLINGER, A.M. & JOHNSON, R.T. (1987). Action of etoposide (VP-16-123) on human cells: no evidence for topoismerase II involvement in excision repair of UV-induced damage, nor for mitochondrial hypersensitivity in ataxia telangiectasia. *Carcinogenesis*, **8**, 1613.

DOWNES, C.S. & JOHNSON, R.T. (1988). DNA topoisomerases and DNA repair. *BioEssays*, **8**, 179.

EARNSHAW, W.C., HALLIGAN, B., COOKE, C.A., HECK, M.M.S. & LIU, L.F. (1985). Topoisomerase II is a structural component of mitotic chromosome scaffolds. *J. Cell Biol.*, **100**, 1706.

ENG W.-K., FAUCETTE, L., JOHNSON, R.K. & STERNGLANZ, R. (1988). Evidence that DNA topoisomerase I is necessary for the cytotoxic effects of camptothecin. *Molec. Pharmacol.*, **34**, 755.

FERTIL, B. & MALAISE, E.P. (1981). Inherent cellular radiosensitivity as a basic concept for human tumour radiotherapy. *Int. J. Radiat. Oncol. Biol. Phys.*, **7**, 621.

FLEISCHMANN, G., PFLUGFELDER, G., STEINER, E.K. & 4 others (1984). Drosophila DNA topoisomerase I is associated with trancriptionally active regions of the genome. *Proc. Natl Acad. Sci. USA*, **81**, 6958.

GALLO, R.C., WHANG-PENG, J. & ADAMSON, R.H. (1971). Studies on the antitumour activity, mechanism of action, and cell cycle effects of camptothecin. *J. Natl Cancer Inst.*, **46**, 789.

GARG, L.C., DI ANGELO, S. & JACOB, S.T. (1987). Role of topoisomerase I in the transcription of supercoiled rRNA gene. *Proc. Natl Acad. Sci. USA*, **84**, 3185.

HECK, M.M.S., HITTELMAN, W.N. & EARNSHAW, W.C. (1988). Differential expression of DNA topoisomerases I and II during the eurkarytoic cell cycle. *Proc. Natl Acad. Sci. USA*, **85**, 1086.

HOLM, C., GOTO, T., WANJ, J.C. & BOTSTEIN, D. (1985). DNA topoisomerase II is required at the time of mitosis in yeast. *Cell*, **41**, 553.

HSIANG, Y.-H., HERTZBERG, R., HECHT, S. & LIU, L.F. (1985). Camptothecin induces protein-linked DNA breaks via mammalian DNA topoisomerase I. *J. Biol. Chem.*, **260**, 14873.

JAXEL, C., KOHN, K.W., WANI, M.C., WALL, M.E. & POMMIER, Y. (1989). Structure-activity study of the actions of camptothecin derivatives on mammalian topoisomerase I: Evidence for a specific receptor site and a relation to antitumour activity. *Cancer Res.*, **49**, 1465.

JOHNSTONE, A. & MCNERNEY, R. (1985). Changes in topoisomerase I activity after irradiation of lymphoid cells. *Bioscience Reports*, **5**, 907.

KELLAND, L.R. & STEEL, G.G. (1988). Modification of radiation dose-rate sparing effects in a human carcinoma of the cervix line by inhibitors of DNA repair. *Int. J. Radiat. Biol.*, **54**, 229.

MATTERN, M.R. & PAINTER, R.B. (1979). Dependence of mammalian DNA replication on DNA supercoiling. II. Effects of novobiocin on DNA synthesis in Chinese hamster ovary cells. *Biochim. Biophsy. Acta*, **563**, 306.

MATTERN, M.R. & SCUDIERO, D.A. (1981). Dependence of mammalian DNA replication on DNA supercoiling. III. Characterisation of the inhibition of replicative and repair-type DNA synthesis by novobiocin and nalidixic acid. *Biochim. Biophys. Acta*, **653**, 248.

MATTERN, M.R., MONG, S.-M., BARTUS, H.F., MIRABELLI, C.K., CROOKE, S.T. & JOHNSON, R.K. (1987). Relationship between the intracellular effects of camptothecin and the inhibition of DNA topoisomerase I in cultured L1210 cells. *Cancer Res.*, **47**, 1793.

NELSON, W.G., LIU, L.F. & COFFEY, D.S. (1986). Newly replicated DNA is associated with DNA topoisomerase II in cultured rat prostatic adenocarcinoma cells. *Nature*, **322**, 187.

NISHIO, A. & UYEKI, E.M. (1982). Inhibition of DNA synthesis in permeabilized L cells by novobiocin. *Biochem. Biophys. Res. Comm.*, **106**, 1448.

ROWE, T.C., WANG, J.C. & LIU, L.F. (1986). *In vivo* localization of DNA topoisomerase II cleavage sites on Drosophila heat shock chromatin. *Mol. Cell. Biol.*, **6**, 985.

SNYDER, R.D. (1987). Is DNA topoisomerase involved in the UV excision repair process? New evidence from studies with DNA intercalating and non-intercalating anti-tumour agents. *Photochem. Photobiol.*, **45**, 105.

STEEL, G.G. & PEACOCK, J.H. (1989). Why are some human tumours more radiosensitive than others? *Radiother. Oncol.*, **15**, 63.

UEMERA, T., OHKURA, H., ADACHI, Y., MORINO, K., SHIOZAKI, K. & YANAGIDA, M. (1987). DNA topoisomerase II is required for condensation and separation of mitotic chromosomes in S. pombe. *Cell*, **50**, 917.

WANG, J.C. (1985). DNA topoisomerases. *Ann. Rev. Biochem.*, **54**, 665.

WANI, M.C., NICHOLAS, A.W. & WALL, M.E. (1986). Plant antitumour agents. 23. Synthesis and antileukaemic activity of camptothecin analogues. *J. Med. Chem.*, **29**, 2358.

WARTERS, R.L., LYONS, B.W., KENNEDY, K. & LI, T.M. (1989). Topoisomerase activity in irradiated mammalian cells. *Mutat. Res.*, **216**, 43.

WILKINS, R.J. (1983). Failure of the intercalating agent m-AMSA to induce DNA repair replication in cultured mammalian cells. *Mutat. Res.*, **122**, 211.

YANAGIDA, M. & WANG, J.C. (1987). Yeast topoisomerases and their structural genes. In: *Nucleic Acids in Molecular Biology, Volume 1*, Eckstein, F. & Lilley, D.M.J. (eds). p. 196. Springer-Verlag: Berlin.